# Preparation of RDX/F2311/Fe_2_O_3_/Al Composite Hollow Microspheres by Electrospray and Synergistic Energy Release during Combustion between Components

**DOI:** 10.3390/ma17071623

**Published:** 2024-04-02

**Authors:** Zhenwei Zhang, Dong Jiang, Lanting Yang, Wenkui Song, Ruihao Wang, Qiuan Huang

**Affiliations:** 1 Co-Innovation Center for New Energetic Materials, Southwest University of Science and Technology, Mianyang 621010, China; zhenwei1219@126.com (Z.Z.); 18908209417@163.com (D.J.); lanting0258@163.com (L.Y.); zhen121819@163.com (W.S.); 2 Automation Research Institute Co., Ltd. of China South Industries Group Corporation, Mianyang 621000, China

**Keywords:** functional composites, thermal properties, enhanced combustion, surface coating

## Abstract

Nanothermites and high-energy explosives have significantly improved the performance of high-energy composites and have broad application prospects. Therefore, in this study, RDX/F2311/Fe_2_O_3_/Al composite hollow microspheres were successfully prepared utilizing the electrospray method using F2311 as a binder between components. The results show that the combustion time of the composite hollow microspheres is shortened from 2400 ms to 950 ms, the combustion process is more stable, and the energy release is more concentrated. The H50 of the composite hollow microspheres increased from 14.49 cm to 24.57 cm, the explosion percentage decreased from 84% to 72%, and the sensitivity of the composite samples decreased significantly. This is mainly the result of the combination of homogeneous composition and synergistic reactions. The combustion results show that F2311 as a binder affects the tightness of the contact between the components. By adjusting its content, the combustion time and the intensity of the combustion of the composite microspheres can be adjusted, which provides a feasible direction for its practical application.

## 1. Introduction

The combustion performance of high-energy explosives has always been a topic of great interest among researchers. As one of the most frequently used explosives in advanced propulsion systems [1,2], 1,3,5-trinitro-1,3,5 triazine (RDX) has the advantages of higher energy, a large amount of gas produced by the reaction, high safety (difficult to ignite at room temperature), low manufacturing cost, and, in particular, the reaction of RDX is also environmentally friendly as it is smokeless, non-toxic, and does not produce corrosive products. In recent years, research on RDX and RDX composites has garnered significant attention in both military and civilian applications [3]. Nano-energetic materials like nanothermite have found widespread use in various civil and military applications, including gas generators [4], electric igniters [5,6], and high-energy additives in explosives and propellants [7,8]. This is due to their rapid oxidation reactions, releasing a substantial amount of heat in a short timeframe.

The biggest advantage of nanothermite is its larger specific surface area and closer contact between components, which shortens the ignition delay period, increases the ignition rate, and accelerates the reaction rate [9,10]. At the same time, nanothermite is also an environmentally friendly energy-containing material, but the small size of the nanopowder itself can lead to an agglomeration phenomenon leading to insufficient combustion [11], and the deficiency in the reaction without gas production affects the application of nanothermite. Combining the respective advantages and disadvantages of fuel and nanothermite, the preparation of the two complexes allows synergistic effects on the reaction to give full play to their advantages [12], but the physical mixing of fuel and nano-alumina thermite is non-homogeneous, resulting in heat and mass transfer being affected by the non-homogeneous reaction kinetics. Hence, with an additive enabling the two to be in close contact, Gregory Young [13] successfully prepared Al/RDX/NC complexes using NC as a binder with reduced ignition delay time of explosives. Ling Chen [14] successfully prepared RDX@CNFs high-energy composites using cellulose nano fibers (CNF), which made the combustion process of the complexes stable. Jian Yao [15] successfully prepared spherical RDX/F2604 using F2604, which improved the thermal and mechanical properties of the complex. It is evident that propellant preparations involving thermite through physical mixing may lead to challenges in maintaining homogeneity, limiting the full potential of thermite’s advantages.

The generally used preparation methods include blocking reaction milling [16,17], electrostatic directional assembly [18,19], sol–gel synthesis [20,21], and electrospray [22,23], which was found to be an effective method for preparing tight complexes about fuel and nano-aluminum thermite in some recent studies. When Tao Yan [24] used electrospray technology to create a composite hollow microsphere out of Al, RDX, and Viton, the results revealed that the composite sample was able to increase the contact area between the components, lower the apparent activation energy of the specimen, and have a quicker ignition delay period and a more intense combustion flame than the physically mixed sample. Lei Xiao [25] prepared Al/CuO/PVDF/RDX composite microspheres by electrospray and showed a significant improvement in both the thermal and combustion properties of the samples. Hongtao Yang [26] prepared Fe_2_O_3_/Al/RDX/NC samples with different RDX contents, and the results showed that the RDX content affected the combustion properties of the composite samples.

It is evident from previous studies that most of the electrospray studies have focused mainly on nano-Al thermal agents and fuels and have not investigated the effect of binder content on the combustion performance of the complexes. Therefore, in this study, RDX/F2311/Fe_2_O_3_/Al microspheres were prepared utilizing the electrospray method. The recrystallized RDX and F2311 (a kind of fluorine rubber series) were intertwined to form hollow microspheres. Fe_2_O_3_ and Al adhered to the hollow microspheres of RDX/F2311 to form composite hollow microspheres. The main reason for using F2311 as the binder is that the pre-ignition reaction between Al_2_O_3_ and F2311 can etch the alumina shell layer indicated by nano-Al to eliminate the adverse effect of Al_2_O_3_ on combustion and expose the aluminum core for reaction with the other components in the composite sample [27,28]. The morphology of the composite sample was characterized by SEM, the composition of the composite microspheres was analyzed by FT-IR and X-ray diffraction, the sensitivity properties of the complexes were tested, and the effect of different contents of F2311 on the combustion properties of the composite microspheres was tested by high-speed photography.

## 2. Experimental Section

### 2.1. Materials

RDX (average particle size of 50 µm) was synthesized in our institute (RDX > 99%). Al NPs (average particle size of 100 nm) were purchased from Shanghai Inagua Materials Technology Co. (Shanghai, China).

α-Fe_2_O_3_ NPs (mass 99.5%, average particle size 30 nm) were spherical and produced from Shanghai Maclean Biochemical Co., Ltd. (Shanghai, China).

F2311 (copolymer of vinylidene fluoride and chlorotrifluoroethylene mixed at a molar ratio of 1:1), 66% fluorine content, was obtained from Zhonghao Chenguang Chemical Research Institute, (Zigong, China).

The solvent was analytical grade acetone, which was supplied by Chengdu Kolon Chemical Co. (Chengdu, China).

### 2.2. Precursor Preparation

First, 140 mg of RDX was weighed and dissolved in a beaker containing 10 mL of acetone. The MIC (metastable intermixed composites) (RDX:MIC = 7:3) materials of 15 mg of Al and 45 mg of Fe_2_O_3_ (Fe_2_O_3_:Al = 3:1) were weighed and placed in a beaker. After 1 h of ultrasound, the precursor solution was successfully prepared by magnetic stirring for 24 h under normal temperature and pressure.

### 2.3. RDX/F2311/Fe_2_O_3_/Al Composite Microsphere Preparation

**Figure 1:**
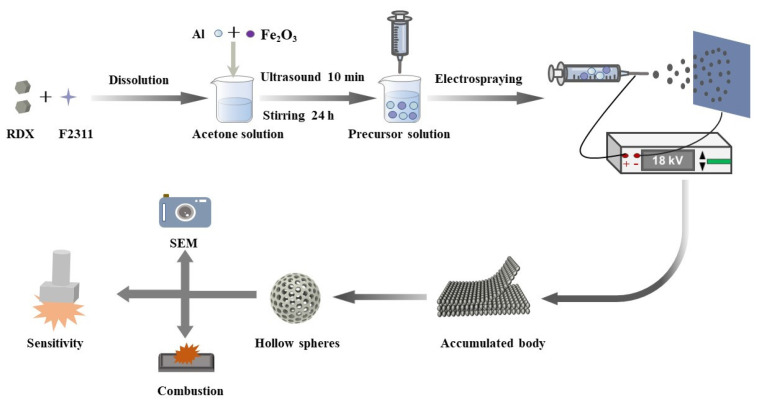
Experimental design preparation flow chart.

The prepared precursor solution was placed into a syringe with an inner diameter of 0.5 mm (21 G), the syringe was fixed to the syringe pump, the power supply voltage of the device was set to 18 kV, the syringe pump advance rate was 0.1 mm/min, and the composite material was collected on an aluminum foil collection plate 15 cm from the needle port. The experimental design principle and preparation process are shown in Figure 1.

### 2.4. Characterization

The morphology of the samples was characterized by scanning electron microscopy (SEM) measurements using an Ultra-55 microscope (Carl Zeiss, Oberkochen, Germany). The compositional analysis of the samples in Fourier transform infrared (FT-IR) spectra was obtained using a Bruker Tensor 27 (BRUKER, Saarbrucken, Germany) in the range of 400–4000 cm^−1^; X-ray diffractograms (XRD, X Pert Pro, PANalytical B.V., Almelo, The Netherlands) were obtained in Bruker D8-Advance diffractometer apparatus at Cu Kα radiation (λ = 0.15405 nm) with a scan range, scan step size, and time per step of 3–80°, 0.03°, and 10.16° for data collection, respectively.

## 3. Results and Discussion

### 3.1. Morphological Analysis

**Figure 2:**
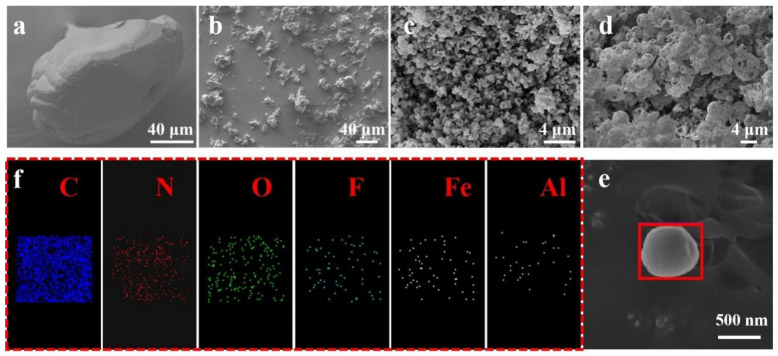
(**a**) RDX raw material; (**b**) RDX prepared by electrostatic spray; (**c**) RDX/F2311 prepared by electrostatic spray; (**d**,**e**) RDX/F2311/Fe_2_O_3_/Al composite microspheres; (**f**) EDS spectra of composite microsphere surfaces.

Figure 2 displays SEM and EDS images of raw RDX, RDX prepared by electrostatic spray, RDX/F2311, and RDX/F2311/Fe_2_O_3_/Al composite microspheres at different magnifications. From Figure 2b,c, it can be found that the addition of F2311 to the components has a facilitating effect on the spherification of the recrystallized RDX. The SEM image in Figure 2c,d shows that F2311 acts as a binder, which aggregates the formed composite hollow microspheres together in layers and maintains the original microsphere structure during aggregation. Figure 2e shows a single composite microsphere size of around 500 nm, and the results of EDS energy spectra performed on the single composite microsphere in Figure 2e are shown in Figure 2f. The composite microspheres contain six elements: C, N, O, F, Al, and Fe, and it can be tentatively assumed that RDX, Fe_2_O_3_, and Al are successfully compounded under the action of F2311.

### 3.2. Compositional Analysis

**Figure 3:**
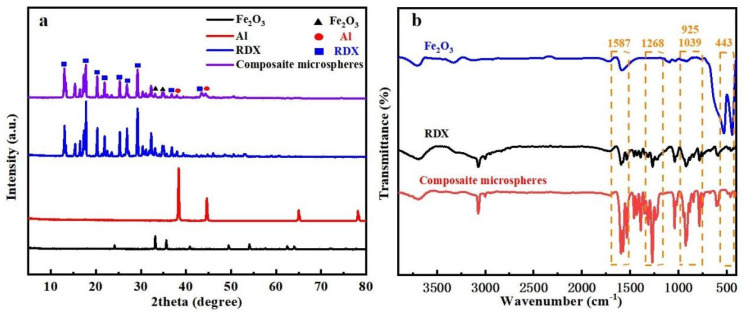
(**a**) XRD patterns of RDX/F2311/Fe_2_O_3_/Al, RDX, Al and Fe_2_O_3_ (**b**) FT-IR spectra of RDX, Fe_2_O_3_ and RDX/F2311/Fe_2_O_3_/Al in the range of 400–3900 cm^−1^.

X-ray diffraction was used to characterize the crystal structure of the composite microspheres, and different crystalline components would produce different characteristic diffraction peaks under X-ray irradiation; the characterization results of the composite microspheres are shown in Figure 3a. It can be seen from Figure 3a that the crystal structure of the composite microspheres prepared using the electrospray method did not change during the recrystallization process of RDX, and the unique peaks typical of the raw material RDX can be observed, followed by the appearance of the typical characteristic peaks of Al and Fe_2_O_3_ in the grain lines of the composite microsphere samples. The crystallinity of the diffraction peaks of the microspheres decreased compared to the raw material due to the reduction in micro/nanometer size. FT-IR characterization of the characteristic chemical groups of the material allows direct chemical identification of the nanomaterials by matching the position, shape, and height of the peaks.

Figure 3b shows the infrared spectrum of RDX/F2311/Fe_2_O_3_/Al composite microspheres. It can be found that the typical absorption band of RDX appears at 1274 cm^−1^ in the composite microspheres. The Fe-O characteristic peak of Fe_2_O_3_ and the bending vibration absorption peak of FeO-OH appeared at 443 cm^−1^ and 1587 cm^−1^, which confirms that the characteristic absorption peaks of each component in the composite microspheres exist. In conjunction with electron microscopy and the preceding analyses, the components were successfully prepared in the composite microspheres prepared utilizing electrospray method, and the combustion performance of the composite microspheres will be further analyzed and tested for the next step.

### 3.3. Combustion Performance

A nickel–chromium resistance wire (diameter: 0.25 mm) was used for heating ignition at one end of the suspension Combustion Velocity Testing: The pRDX@PTFE-Al Composite sample is ignited by Cr–Ni alloy silk (diameter: 0.4 mm, length: 3 cm, melting point: 1200 °C) in the open air, which is heated by direct current power supply (MS-3010D, MAISHENG, Dongguan, China) under 7.5 V. The open flame combustion experiments were photographed using high speed photography (Canon, EOSKiss70D, Tokyo, Japan) to study the combustion process, energy output, and the interaction between the components [29]. It can be seen that although RDX is an excellent rocket propellant component, its raw material itself cannot be ignited by open flame [30,31,32] nor by resistance-wire heating at room temperature and atmospheric pressure, so the ignition pre-reaction in the combustion process primarily relies on the energy generated by the thermite reaction as the initial energy for RDX ignition.

In RDX/F2311/Fe_2_O_3_/Al composite microspheres, the amount of Fe_2_O_3_/Al attached to the RDX hollow microspheres affects the amount of Fe_2_O_3_/Al attached to the RDX hollow microspheres, which in turn affects the combustion effect of the composite samples, and since the addition of F2311 in the formulation will react with the shell layer in advance, a physical mixing method was used to compare it. The results showed that the flame of the sample with the addition of F2311 was bright yellow, and the closer to the combustion surface it was, the brighter the flame was, as previously validated by JIAO Y [31]. This effect is attributed to the small amount of F2311 added and the uneven mixing of components in physically mixed samples. So, F2311 first reacts with the Al_2_O_3_ shell layer on the surface of the Al powder to produce AlF_3_ gas. This is also why F2311 measurements are more violent in the early half of the combustion. Due to the reaction between Al powder and F2311, the combustion period is prolonged in the second half, which reduces the heat produced by the Al_2_O_3_ reaction and slows down the combustion speed. From the flame images, the flame edges of the composite samples have a smooth structure of spark sputtering, in contrast to the presence of sputtering of solid particles in the physically mixed samples, which is mainly generated by gaseous products that sputter the incompletely burned Al powder in the components.

**Figure 4:**
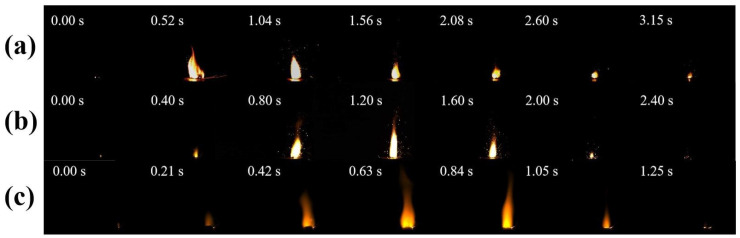
Combustion images of (**a**) Mix-RDX/F2311/Fe_2_O_3_/Al, (**b**) Mix-RDX/Fe2O3/A,l (**c**) electrospray composite microspheres.

Therefore, it can be concluded that for the samples prepared utilizing the electrospray method: the samples are more evenly dispersed in the components, the contact between the components is more compact, the Al powder is completely burned, the flame has been in a stable combustion state throughout the combustion process, and the composite microsphere combustion has a much more violent time and is far faster than that of the mixed sample. The analysis of Figure 4a also proves that the addition of F2311 in the composite sample can help the combustion of aluminum powder, and F2311 can promote the combustion of the composite sample in the case of uniform mixing of components. In terms of the combustion rate, the total combustion time of the physically mixed sample was about 3.15 s, while the total combustion time of the RDX/F2311/Fe_2_O_3_/Al composite microspheres prepared utilizing the electrospray method was about 0.95 s; the combustion rate was more than double that of the physically mixed samples. The advantages of the electrospray method for sample preparation were thus fully demonstrated. The reduction in the overall combustion time can obtain a larger specific impulse and energy in a very short time, which can meet the requirements of the propellant system that needs to be more rapid and violent.

**Figure 5:**
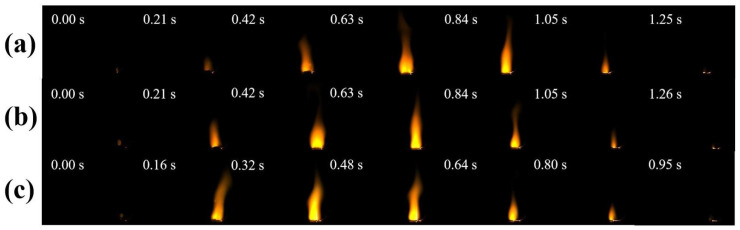
Combustion images of electrosprayed composite microspheres with different F2311 mass ratios: (**a**) 3 wt%-F2311, (**b**) 4 wt%-F2311, (**c**) 5 wt%-F2311.

Therefore, to investigate the effects of F2311 content and nano-Al content on the combustion properties of composite microspheres, the effects of different contents of F2311 and nano-Al on the combustion properties of the composite microspheres were carried out at the mass ratio RDX:(Fe_2_O_3_:Al = 3:1) = 7:3. Figure 5a–c show the combustion of the composite microspheres at F2311 mass ratios of 3 wt%, 4 wt%, and 5 wt% flame images.

**Figure 6:**
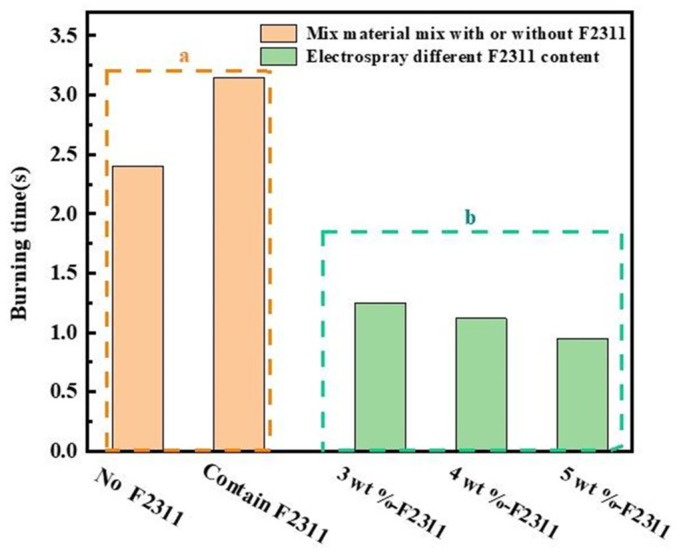
Burning time graph for different samples. (**a**) Physical mixing with or without F2311 and (**b**) F2311 prepared with electrospray with mass fractions of 3 wt%, 4 wt%, 5 wt%.

According to Figure 5a–c, the combustion rate of the composite samples gradually accelerates and reaches the most violent moment of combustion more quickly as F2311 content increases, and when F2311 increases by 1 wt%, the combustion time is shortened by about 150 ms, causing its main reasons to be divided into two cases:

(1) The combustion of conventional nano-alumina powder results in the formation of a dense inert layer on the powder’s surface. This layer hinders the diffusion of oxidizing gases within the powder. Increasing fluorine content leads to the destruction of more of the alumina shell layer, and the sublimation temperature of the aluminum fluoride produced (1276 °C) is lower than the combustion temperature of the nano-alumina. The exposed surface of the aluminum fluoride post-sublimation serves as reactant for further oxidation consequently accelerating the combustion time of the composite microspheres and reaching peak combustion intensity faster.
(2) The scanning electron microscope (SEM) image in Figure 2 reveals a significant number of composite microspheres arranged in stacks. Within these composite microspheres, F2311 serves as a binder that consolidates the other components. Additionally, Figure 6 illustrates the burning times of various samples. A correlation between the F2311 content and burning time is evident, highlighting the advantages of the preparation method. Specifically, an increase in the F2311 content results in closer component contact, shorter heat and mass transfer distances, and ultimately, shorter burning times. Notably, no noticeable difference in combustion duration was observed when the mass fraction of F2311 was raised to 5 wt%.

### 3.4. Sensitivity Characterization

**Figure 7:**
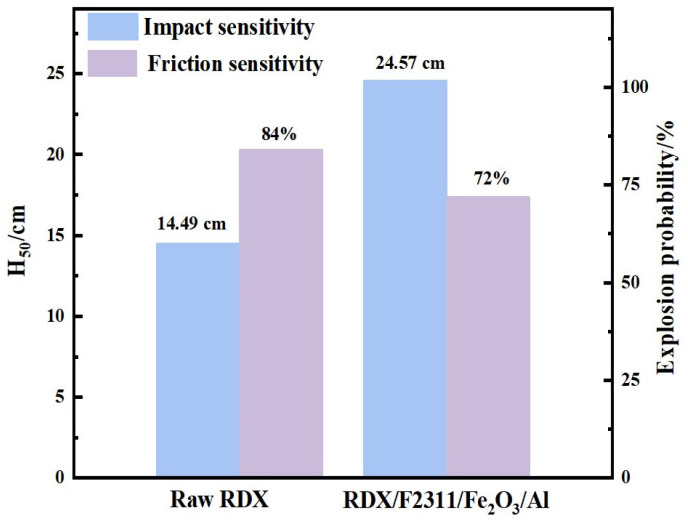
Sensibility tests.

The sensitivity performance is characterized according to Chinese military standards GJB772A-97-601.2 (impact) and 602.1 (friction). The impact sensitivity test conditions are as follows: the weight of the falling weight is 10 kg, the drug dosage is 50 mg, 25 samples are tested, the impact sensitivity is expressed by the height of the falling weight H_50_, and the probability of sample explosion is 50%. The friction sensitivity test conditions are swing angle 90°, pressure 3.92 MPa, drug dose 20 mg; 25 samples are tested, and the friction sensitivity can be expressed in terms of explosion probability. The test results are shown in Figure 7. The H_50_ of raw material RDX is 14.49 cm, and the H_50_ of the composite hollow microspheres is 24.57 cm. The friction sensitivity explosion percentage of the composite microspheres is reduced from 84% of the raw material to 72%. Overall, the impact sensitivity of the composite microspheres has a significant reduction effect, while the friction sensitivity has a certain reduction. This is mainly because impact sensitivity is dominated by hotspot theory, while the mechanism of friction sensitivity is inconsistent with the impact sensitivity mechanism, and the specific mechanism has not yet been clarified. The main reason for the decrease in sensitivity is related to the size reduction in and unique hollow structure of the composite microspheres. When the sample is impacted, the unique hollow structure will reduce part of the impact energy, and the non-energy components in the component can absorb part of the energy, thereby reducing the formation of hotspots and achieving the effect of lowering sensitivity. Therefore, the composite hollow microspheres prepared utilizing the electrospray method can not only effectively improve combustion performance but also reduce sensitivity.

### 3.5. Mechanism Analysis

**Figure 8:**
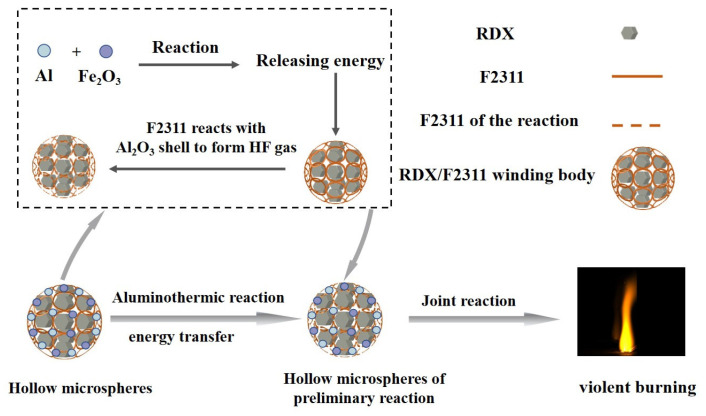
Mechanism of action diagrammatic drawing.

Compared with the raw materials, the combustion performance and sensitivity of the RDX/F2311/Fe_2_O_3_/Al composite hollow microspheres have been significantly improved. The mechanism of action is shown in Figure 8. Due to the Al and Fe_2_O_3_ complex attached to the surface of the RDX/F2311 filament, when stimulated by an external heat source, part of the energy promotes the reaction between Fe_2_O_3_ and Al, while the other part of the energy is transferred to the RDX/F2311 filament, together with the energy generated by the aluminothermic reaction, resulting in the melting of the F2311. The melted F2311 reacts with the alumina shell on the surface of the Al powder to generate AlF_3_ gas, which makes the more active aluminum participate in the reaction and greatly improves the intensity of combustion. Under the action of energy accumulation inside the microspheres, the RDX will also react rapidly, generating energy and gaseous products to further promote the reaction of Fe_2_O_3_ and Al, thereby significantly improving the overall combustion performance, shortening the combustion time and making the energy release more concentrated. This is the result of the synergistic effect between the components and the unique composite hollow sphere.

## 4. Conclusions

In summary, RDX, F2311, Fe_2_O_3_, and Al high-energy hollow microspheres were successfully prepared utilizing the electrospray method. The test results show that compared with the physical mixed samples, the combustion rate of the composite microspheres prepared by electrospraying is greatly improved, the combustion time is shortened by 60%, the combustion process is more stable, the heat release is more concentrated, and the combustion performance is significantly improved. Under the combined action of factors such as sphericity and size reduction, the impact sensitivity H_50_ of the composite hollow microspheres increased from 14.49 cm to 24.57 cm, and the friction sensitivity also decreased to a certain extent. This study not only proves that thermite is an effective supplementary strategy for the combustion performance of RDX but also finds that the combustion time of the composite material can be effectively adjusted by adjusting the content of F2311, which provides an effective strategy for the functional application of RDX in composite propellant systems.

### Matters Needing Attention

This experiment involves dangerous chemicals, including acetone solution and RDX. The dangerous operation steps include the high-pressure use of the electrospray machine during the preparation process, the safety protection in the sensitivity test, etc. Special attention should be paid during the experiments.

## Data Availability

Data are contained within the article.
